# Highly sensitive and accurate detection of C-reactive protein by CdSe/ZnS quantum dot-based fluorescence-linked immunosorbent assay

**DOI:** 10.1186/s12951-017-0267-4

**Published:** 2017-05-02

**Authors:** Yanbing Lv, Ruili Wu, Kunrui Feng, Jinjie Li, Qing Mao, Hang Yuan, Huaibin Shen, Xiangdong Chai, Lin Song Li

**Affiliations:** 1 Key Laboratory for Special Functional Materials of Ministry of Education, Henan University, Kaifeng, 475004 China; 2 NepQD Biotech Corp, Taizhou, 225300 China

**Keywords:** Quantum dots, Fluorescent probe, Fluorescence-linked immunosorbent assay, C-reactive protein

## Abstract

**Background:**

The conventional and widely used enzyme-linked immunosorbent assays (ELISA), due to specificity and high-sensitivity, were suitable in vitro diagnosis. But enzymes are vulnerable to the external conditions, and the complex operation steps limit its application. Semiconductor quantum dots have been successfully used in biological and medical research due to the high photoluminescence and high resistance to photobleaching. In this study, we have developed a novel quantum dot-labeled immunosorbent assay for rapid disease detection of C-reactive protein (CRP).

**Results:**

The assay for the detection of CRP can provide a wide analytical range of 1.56–400 ng/mL with the limit of detection (LOD) = 0.46 ng/mL and the limit of quantification = 1.53 ng/mL. The precision of the assay has been confirmed for low coefficient of variation, less than 10% (intra-assay) and less than 15% (inter-assay). The accuracy of assay meets the requirements with the recoveries of 95.4–105.7%. Furthermore, clinical samples have been collected and used for correlation analysis between this FLISA and gold standard Roche immunoturbidimetry. It shows excellent accurate concordance and the correlation coefficient value (R) is as high as 0.989 (n = 34).

**Conclusions:**

This in vitro quantum dot-based detection method offers a lower LOD and a wide liner detection range than ELISA. The total reaction time is only 50 min, which is much shorter than the commercialization ELISA (about 120 min). All of the results show that a convenient, sensitive, and accurate fluorescence-linked immunosorbent assay method has been well established for the detection of CRP samples. Therefore, this method has immense potential for the development of rapid and cost-effective in vitro diagnostic kits.

**Electronic supplementary material:**

The online version of this article (doi:10.1186/s12951-017-0267-4) contains supplementary material, which is available to authorized users.

## Background

Currently, enzymes as labels have been widely used in commercially quantitative immunoassays [1, 2], especially enzyme-linked immunosorbent assay (ELISA) [3, 4] is frequently used in in vitro diagnosis because of its high sensitivity and specificity [5, 6]. The signal collection in ELISA are based on a change in optical transmittance by a time-dependent enzymatic reaction, but enzymes are usually vulnerable to external conditions and very susceptible to matrix constituent [7]. So these complicated operation steps may limit its application. Compared to ELISA, a new approach called fluorescence-linked immunosorbent assay (FLISA) has been developed recently [8–10]. The emission of fluorescent signal as the analytical signal can be immediately recorded by the microplate reader, and it reduces the analysis time because of no chromogenic reaction steps of ELISA [11].

Due to the excellent optical characteristics [12, 13], Semiconductor quantum dots (QDs) are ideal fluorescent probes for advanced biosensor [14, 15], cellular imaging [16–18], cell labeling [19, 20], gene sequencing [21, 22], and chemical analysis [23, 24]. Thereafter, QD-based fluorescence probes begin to be introduced in the field of in vitro diagnosis [25–27]. Lin and co-workers first integrated QDs into lateral flow immunoassay (LFIA) and used to detect the concentration of nitrated ceruloplasmin [28]. We also adapted LFIA for the detection of human chorionic gonadotrophin (HCG) antigen by using CdSe/ZnS QDs microspheres as fluorescence probes successfully [29]. The quantitative result showed that the sensitivity of HCG antigen detection could reach 0.5 IU L^−1^, which was almost 20 times higher than traditional LFIA using tinctorial labels. Most recently, we have developed a sensitive quantitative LFIA to detect influenza A virus subtypes H5 and H9 simultaneously [30], the accuracy is as high as that of real-time PCR.

As highly environmental-stable, photo-stable, and fluorescence quantum yield materials, QDs are potentially to be used as new generation fluorescence labels in FLISA and can be widely used in in vitro diagnosis [2, 31–33]. Recent developed “green” synthesis of QDs has successfully lower the total cost and provides an opportunity to use QDs as affordable labeling materials [34]. Here in this paper, we have developed a novel quantum dots-based FLISA for disease detection. C-reactive protein (CRP), as an acute phase protein from liver cells, has been used clinically to monitor infection and autoimmune disorders [35, 36]. Especially, high-sensitivity C-reactive protein (hs-CRP), much higher sensitivity than CRP, has been regarded as an independent biomarker for cardiovascular disease (CVD) in clinic [37, 38].

**Scheme 1:**
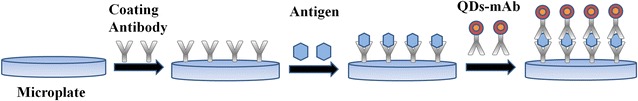
A schematic illustration of FLISA procedure

This FLISA approach employs a novel water-soluble QDs fluorescent probe by using CdSe as core and ZnS as shell, instead of enzyme-linked antibody, and the process is shown in Scheme 1. The high quality CdSe/ZnS core/shell QDs were synthesized according to the “green” phosphine-free and low-cost synthesis method developed by our group [34]. First, CRP antigens were captured by the coating CRP antibodies on the microplate. Then, the labeled antibodies combined to antigens, and a mAb-Ag-mAb sandwich structure was formed after sufficient incubation time. The fluorescence intensity was proportional to the concentration of CRP antigens. The optimal reactive conditions have been found in this study, including the stability of QDs-mAb and optimal incubation time etc. As expected, this novel method has been proven that it can not only shorten the total reaction time, but also simplify the operation steps. And more important, the detection sensitivity and accuracy have been greatly improved. The further development of this immunoassay has immense potential for future convenient and cost-effective in vitro nano-medical diagnostic kits.

## Methods

### Materials and instruments

Cadmium oxide (CdO, 99.99%), zinc oxide (ZnO, 99.99%, power), sulfur (S, 99.98%, power), selenium (Se, 99.99%, power), oleic acid (OA, 90%), 1-octadecene (ODE, 90%), and 2-(*N*-morpholino) ethanesulfonic acid (MES) were purchased from Aldrich. NaOH, HCl, NaCl, KCl, Na_2_CO_3_, NaHCO_3_, KH_2_PO_4_, Na_2_HPO_4_, H_3_BO_3_, Na_2_B_4_O_7_∙10H_2_O, Tris, Hepes, and Tween-20 were purchased from Shanghai Sangon Ltd (China). Bovine serum albumin (BSA) and calf serum were purchased from Sigma. 1-ethyl-3-(3-(dimethylamino) propyl) carbodiimide (EDC), *N*-Hydroxysulfosuccinimide (sulfo-NHS) and the microplates were purchased from Thermo Fisher Scientific (USA). Mouse anti C-reaction protein monoclonal antibody and CRP antigen were obtained from Abcam (USA). The fluorescence spectra were detected using SpectraMaxi3 (Molecular Devices, Sunnyvale, USA). Images of electrophoresis gels were taken using a gel imaging system (GenoSens1860, Shanghai, China). The sizes of QDs and QD-antibody probe were recorded using dynamic light scattering (Nano-ZS 90, Malvern Instruments, UK). Purified water (18.2 mΩ, Millipore USA) was used in all experiments.

### Synthesis of hydrophobic CdSe/ZnS QDs and aqueous CdSe/ZnS QDs

The synthesis of CdSe/ZnS core/shell QDs was performed according to the previous literature from our group [34]. The PL and absorption spectra of the hydrophobic QDs are shown in Additional file 1: Figure S1. Amphiphilic oligomers (polymaleic acid n-hexadecanol ester, PMAH) were chosen as surfactants to prepare aqueous CdSe/ZnS QDs in this work [29, 39]. The hydrophobic chains of amphiphilic oligomers were used to combine the hydrophobic coatings on the CdSe/ZnS QDs and free carboxylic acid groups were available for further surface modification. The CdSe/ZnS QDs could be transferred into water by a phase transfer method in Additional file 1: Figure S2 (detailed operations in supporting information).

### Preparation of QDs-antibody detecting probes

**Scheme 2:**
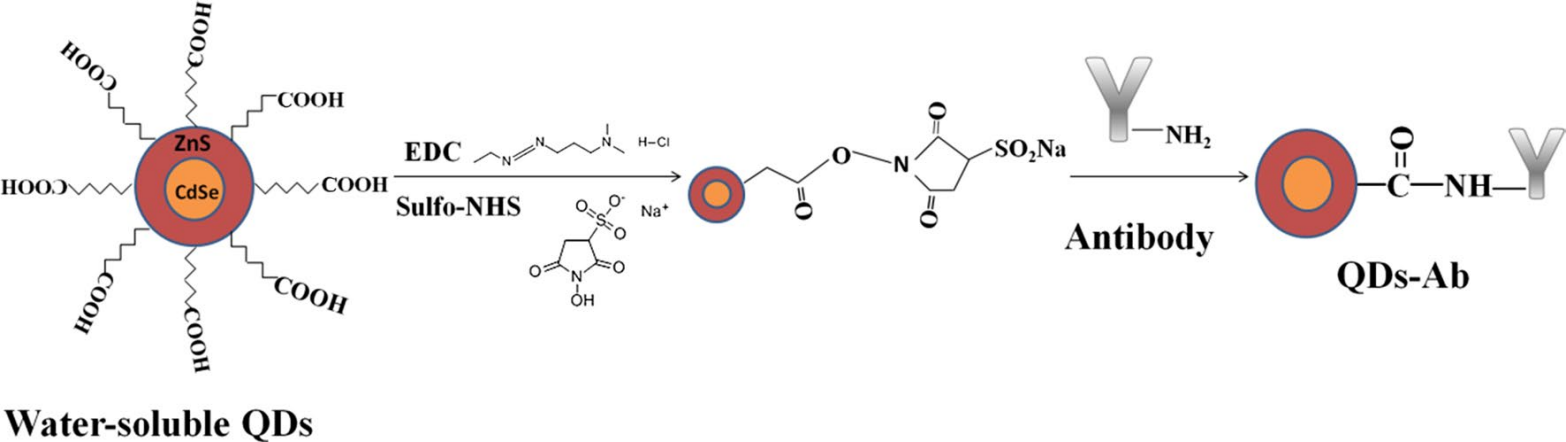
Formation of QD labeled antibody

The QDs were conjugated with monoclonal antibody (shown in Scheme 2) according to the following protocol: first, 150 μL of PMAH-coated aqueous QDs (5 mg/mL) dissolved in 750 μL sodium borate buffer (5 mM, pH 7.2, BS buffer), then it was mixed with 50 μL of suflo-NHS (49 mg/mL in 5 mM BS buffer, pH 7.2) and 50 μL of EDC (17.36 mg/mL in 5 mM MES buffer, pH 5.5). The mixture was activated to react at 4 °C for 5 min under ultrasonic. Therewith, the superstratum clear-solution was abandoned after centrifuge separation at 20,000 rpm and 4 °C for 30 min. Second, 27.6 μL monoclonal antibodies (2.5 mg/mL) were added into the QDs with dissolving in 200 μL BS buffer (5 mM, pH 9.0) and incubated at 37 °C for 2 h. Afterwards, the mixture was blocked by 1% BSA solution and incubated at 37 °C for 30 min. Finally, the probe was washed by 5 mM BS buffer (pH 8.0). The QDs-mAb was stored in 50 μL Tirs solution (5 mM, pH 8.0) for future use.

### Preparation of antibody coated fluorescence microplate

Primary antibody (the concentration of CRP monoclonal antibody was 1.8 mg/mL) in every well of microplate was diluted with a carbonate–bicarbonate buffer (50 mM, pH 9.6, CB buffer). Subsequently, the microplate was covered with sealing film and incubated at 4 °C for 24 h. In order to remove extra coating antibody, the microplate was washed three times with a wash buffer (0.05% Tween-20 in 10 mM PBS, pH 7.4). Then excess binding sites were blocked with 0.5% (w/v) BSA in 10 mM PBS (pH 7.4) for incubating overnight at 4 °C, this process ensured that all the available and remaining binding sides of the microplate wells were covered. The microplate was dried in a constant temperature humidity chamber for 24 h, then it was stored at 4 °C until use.

### Optimization of reaction condition

In order to obtain the optimum conditions, various analytical conditions have been studied, including the screening QD–mAb probe diluted buffer (detailed content provided in Additional file 1: Figure S4), the optimization of reaction time, the coated primary monoclonal antibody concentration and the dilution ratio of the QDs-mAb probe. Then, under the optimization of reaction condition, an ideal regression coefficient for the standard curve has been obtained.

### Quantitative detection of the CRP standard antigens by fluorescence-linked immunoassay

In every well of a 96-well microplate that contained coating antibody, 100 μL of the standard antigens, diluted to a series of concentrations with the sample buffer (10% calf serum (v/v) in 0.1 M PBS), were incubated at 37 °C for 30 min. Then the plate was followed by five washes with a wash buffer (PBS-T) at 1 min per wash. For subsequent operation, added 100 μL of the QDs-mAb probes diluted with the probe buffer (10% calf serum (v/v) in 0.1 M PBS) into each well were incubated at 37 °C for 30 min. After five washes with a wash buffer (PBS-T), the fluorescence intensity of each well in the plates was automatically read out by SpectraMax i3 when 450 nm was used as excitation wavelength.

### Analysis of human serum samples

A total of 34 CRP human serum samples were obtained from Shenzhen Sixth People’s Hospital. Roche immunoturbidimetry assay was also utilized as a reference method to assess the accuracy of FLISA. Analysis samples were mixed with 1 μL human serum samples and 399 μL the sample buffer (the human serum samples were diluted 400 times). Each sample was tested three times respectively, and the mean value of the results was chosen.

## Results and discussion

### Characterization of QDs-mAb probes

**Fig. 1:**
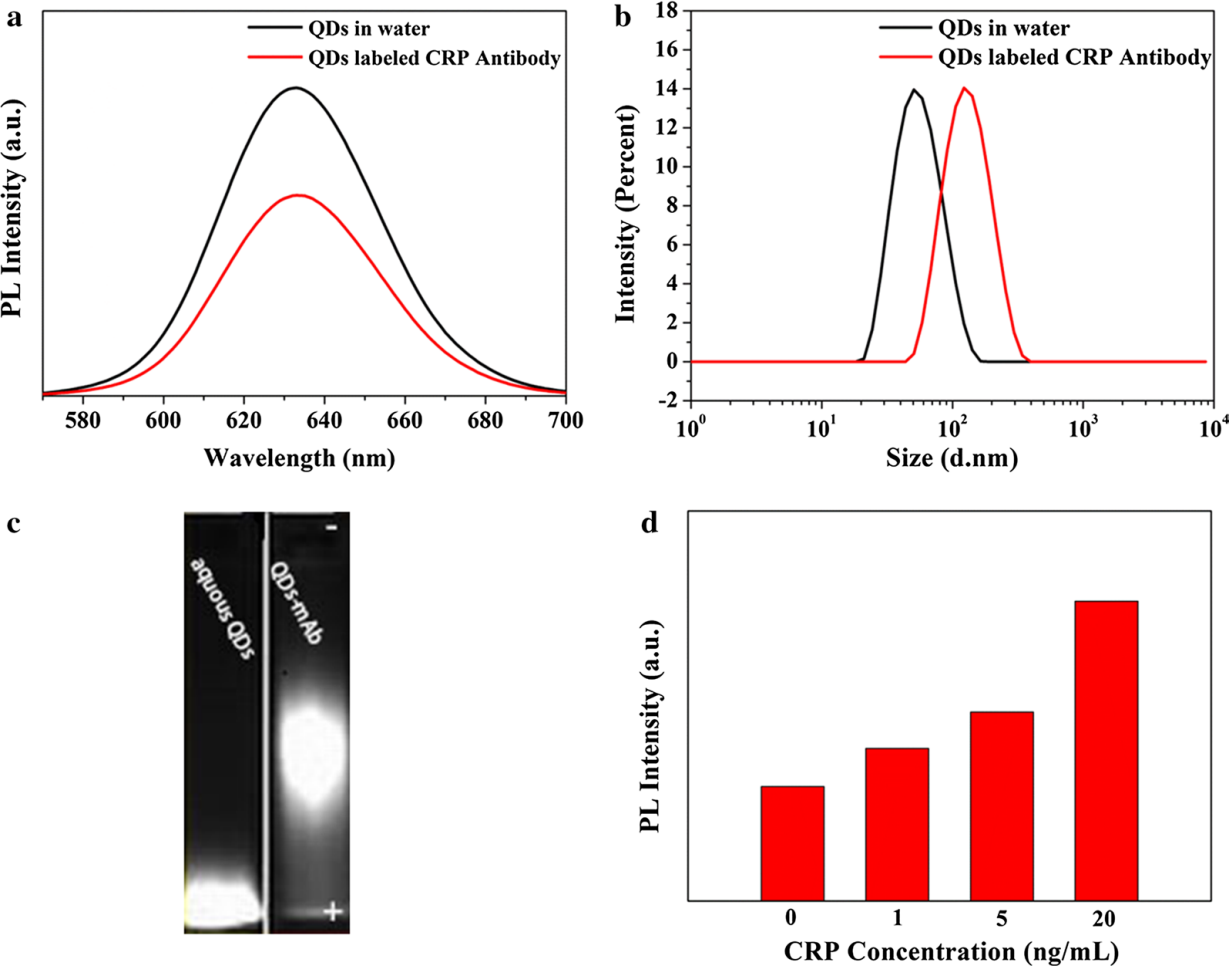
Fluorescence spectra (**a**), dynamic light scattering (**b**), and agarose gel electrophoresis imaging (**c**) of the aqueous CdSe/ZnS QDs and QDs-mAb; and PL intensity of FLISA with different antigen concentrations (**d**)

Fluorescence spectra of aqueous CdSe/ZnS QDs and the QDs-mAb have been collected, the results are shown in Fig. 1a. Generally, the PL peak position of high quality CdSe/ZnS core/shell QDs is mainly determined by the band gap of CdSe, and it is not affected by its hydrodynamic sizes and ligands. Although the hydrodynamic size of QDs is increased after labeled with CRP antibodies (shown in Fig. 1b), the fluorescence peaks were approximately no changes on peak position and peak shape, and kept symmetrical in both aqueous QDs and QDs-mAb solutions. But the fluorescence intensity was declined to 64% on account of centrifuge separation during the coupling process.

In order to investigate the effect of conjugation on the size of QDs, the aqueous QDs and QDs-mAb were characterized by dynamic light scattering and agarose gel electrophoresis. The dynamic light scattering result clearly shows the narrow and uniform size distribution in Fig. 1b, and the hydrodynamic size of QDs increases from 50 to 120 nm after the conjugation with CRP antibodies, which can suggest the successful formation of QDs-mAb. The agarose gel electrophoresis (shown in Fig. 1c) indicates that the QDs-mAb probe moves slower than the aqueous QDs, due to the larger size of the QDs-mAb probe compared to the aqueous QDs, which means CRP antibodies have been successfully connected. Generally, zeta potential analysis can be used to evaluate that the stability of colloids, the value above +30 mV, or below −30 mV is believed to be stable in water. The zeta potential of the aqueous CdSe/ZnS QDs and QDs-mAb was measured and shown in Additional file 1: Figure S3. After the antibody conjugation with aqueous QDs, zeta potential is only changed from −46.8 to −40.0 mV, which is still stable in water. Figure 1d obviously illustrates that the fluorescence intensity increases with the increasing of antigens concentration (the red column diagram). It turned out that the aqueous QDs and CRP antibody protein maintained the original activity after the coupling process. The admirable stability of the aqueous QDs can be applied to coupling of other antibody protein as fluorescent probe.

**Fig. 2:**
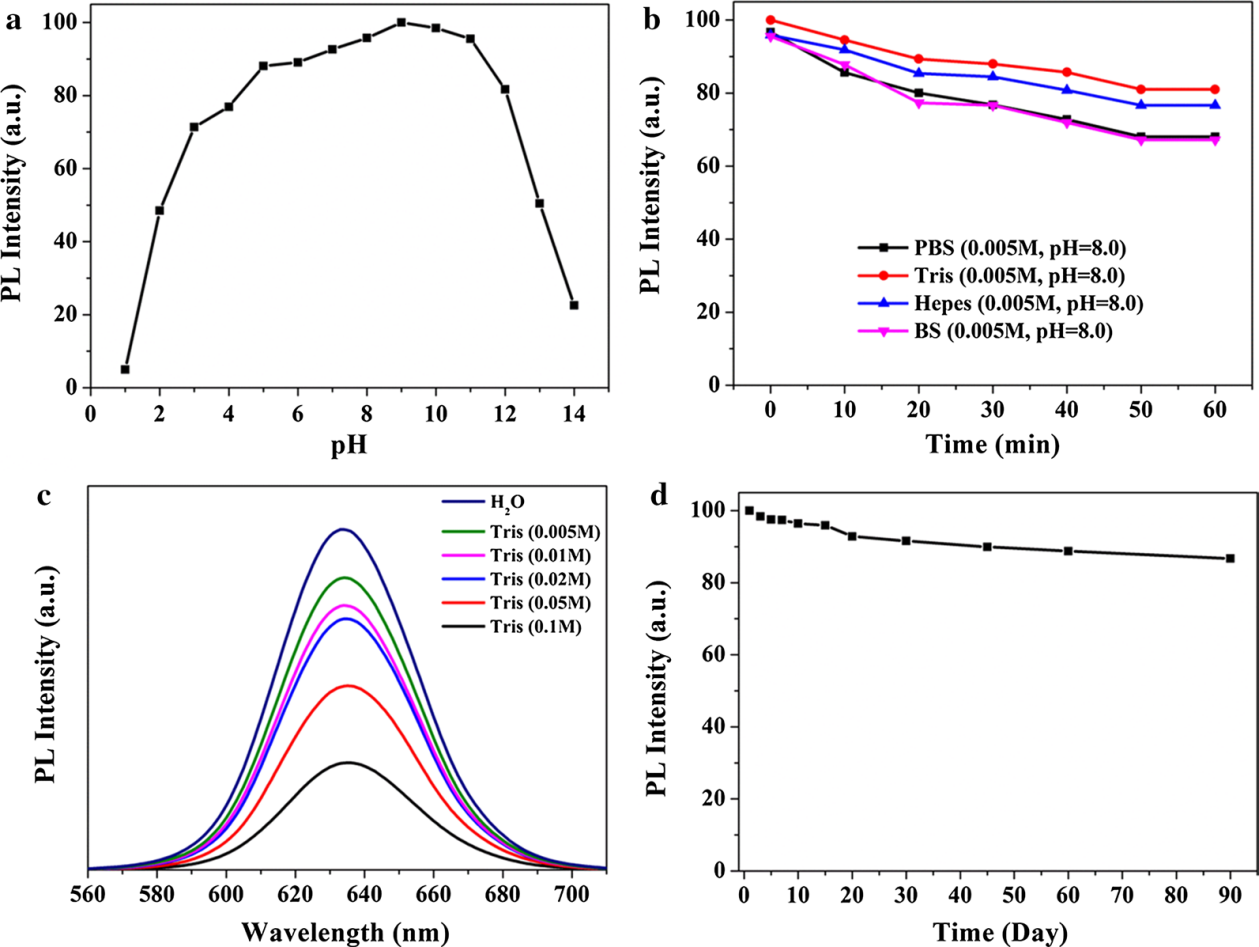
The PL intensity of QDs-mAb in various pH (**a**) and in different buffers (**b**). Fluorescence spectra of QDs-mAb in Tris buffer solutions with different ionic strengths (**c**). The PL intensity of QDs-mAb probes versus different storage time (**d**) in Tris buffer solution

The stabilities and optical properties of QDs-mAb probe were examined and compared in physiological relevant environmental conditions. The PL intensity values of QDs-mAb in acidic-to-alkaline pH environments were measured in following experiment (Fig. 2a). QDs-mAb was stable in a wide range of pH (4–12), the fluorescence intensity was able to be kept >75% even after 1 h preservation. The QDs-mAb was diluted into different buffers with 1 h preservation at 37 °C, the fluorescence intensities were measured (0, 10, 20, 30, 40, 50, 60 min, respectively). As shown in Fig. 2b, the effect of different buffers on the fluorescence stability of QDs-mAb show that the highest fluorescence intensity of the QDs-mAb could be kept in Tris buffer. Subsequently, the influence of fluorescence intensity with Tris buffer (pH 8.0) at different ionic strengths was studied (shown in Fig. 2c). It indicated that the fluorescence intensity was increased with the decreasing of the concentration. The QDs-mAb in Tris buffer (5 mM pH 8.0) showed the maximum fluorescence intensity, which was chosen as the storage buffer for the following experiments. The result shown in Fig. 2d illustrated that the storage stability was studied by measuring the fluorescence spectra of QDs-mAb in Tris buffer for a period of time (stored in 4 °C). The fluorescence intensity decreased to 86.7% after three months storage, which explicitly instructed the novel QDs-mAb could be applied to industrial production due to the excellent stability.

### Chessboard titration to determine the best proportion of coated antibody and label antibody

To confirm the optimum conditions of coating antibody concentration and the dilution ratio of QDs-mAb probe, we designed a chessboard titration experiment (Table 1). The coating antibody was diluted to different concentrations (10, 5, 1, 0.1 μg/mL) with CB buffer, and 100 μL of various coating antibody solutions were fixed in microplate. Subsequently added different concentration antigen (0, 1, 50 ng/mL) in corresponding wells and incubated for 30 min at 37 °C. The diluted ratio was 1:100, 1:200, 1:300, 1:400 (v/v) with the probe buffer of the QDs-mAb, followed by incubating for 30 min at 37 °C. The optimum condition was chosen from higher fluorescence intensity, and it is that the concentration of coating monoclonal antibody was 5 μg/mL when the diluted ratio of QDs-mAb was 1:100.

**Table 1:** Optimization of working concentration

Coating antibody		10 μg/mL			5 μg/mL			1 μg/mL			0.1 μg/mL		
C_Ag_ (ng/mL)		0	1	50	0	1	50	0	1	50	0	1	50
D	1:100	33,506	39,216	614,441	30,142	35,891	640,496	27,993	41,092	635,737	32,337	29,902	40,606
	1:200	27,232	36,745	495,871	29,246	36,645	619,561	25,237	32,604	507,215	28,922	27,300	36,226
	1:300	26,143	31,833	344,716	27,453	34,398	427,663	22,174	33,261	388,885	27,790	27,253	31,046
	1:400	25,002	31,137	240,503	27,130	30,330	247,904	22,373	32,398	192,264	28,985	28,918	29,168

*D* The dilution ration of QDs-mAb probe

### Optimization of incubating time

**Fig. 3:**
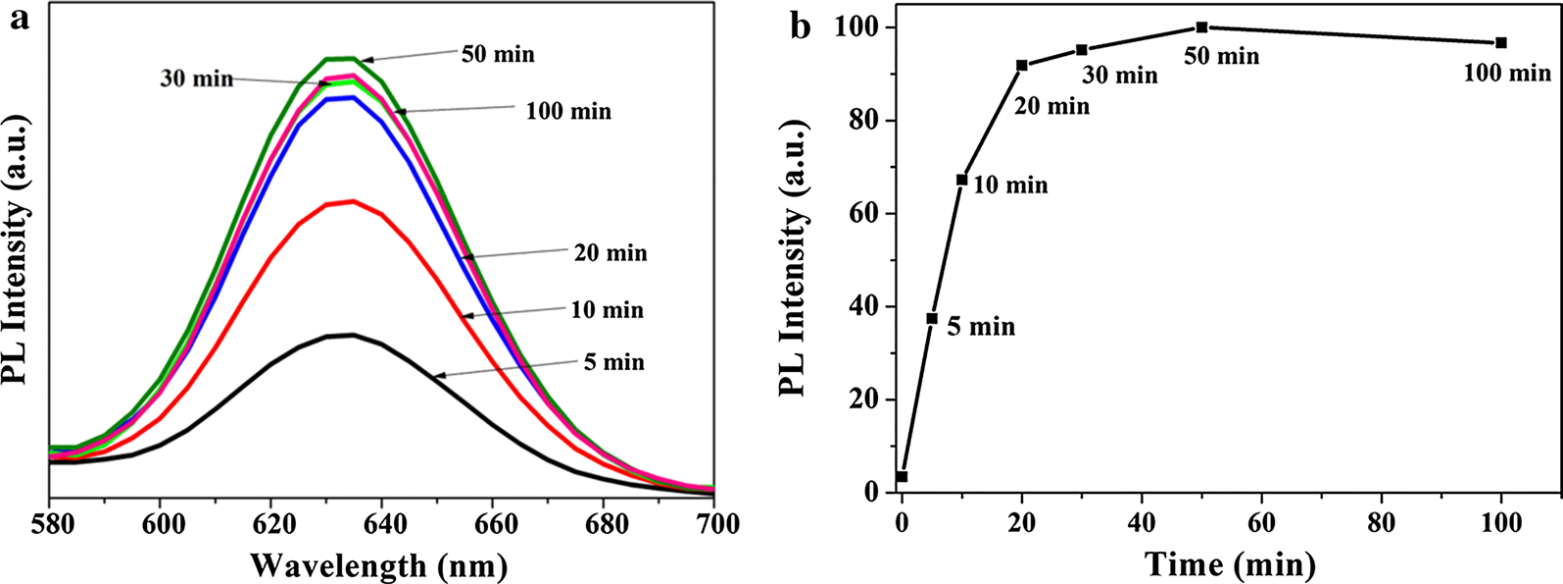
Evolution of PL intensity in different fluorescence developing time (**a**) and optimal fluorescence developing time (**b**)

In order to optimize the fluorescence developing time in detection, the fluorescence intensity was detected in different developing time. Here we changed the incubating time of QDs-mAb probe after the CRP antigen (50 ng/mL) was incubated for 30 min. The results shown in Fig. 3a and b indicate that the optimum fluorescence developing time was 20 min, when a dynamic balance was reached between the coating mAb-Ag and labeled-mAb reaction. However, the max fluorescence intensity at 50 min began to decrease because the label antibodies tend to be decomposed from the mAb-Ag-labeled mAb compounds. Thereof we selected 20 min as the optimum reaction because it shortened the reaction time under the premise of guarantee the fluorescence intensity. In a word, this quantum dot-labeled immunosorbent assay method controlled the reactive time within 50 min, which shortened the reaction time over 70 min comparing to the commercialization ELISA (the reactive time about 120 min).

### Calibration curves and linearity of analytical results

**Fig. 4:**
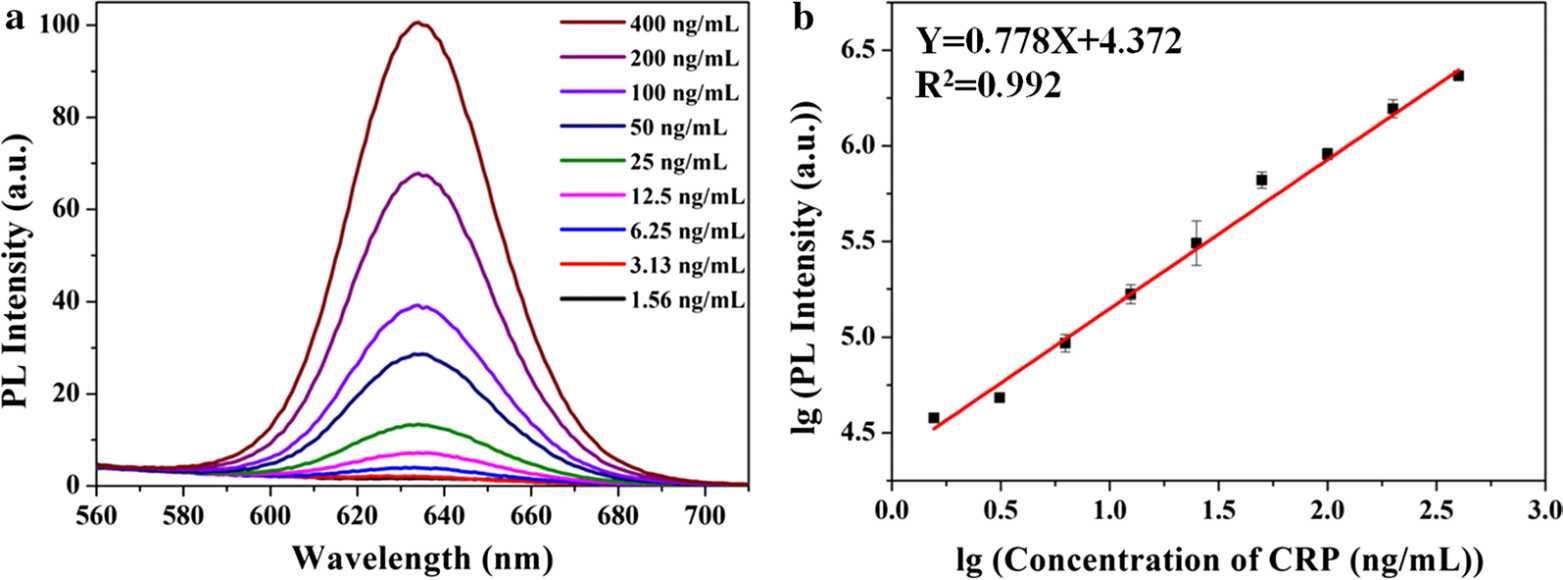
Photoluminescence spectra from FLISA for determination of CRP antigen (**a**) and standard curves (**b**) (n = 3)

In the assay, standard CRP antigen (20 μg/mL) was diluted to 1.56, 3.125, 6.25, 12.5, 25, 50, 100, 200, and 400 ng/mL with the sample buffer, then followed to be incubated and washed by the washing buffer. The QDs-mAb probe was added into corresponding wells of microplate after dilution, the fluorescence intensity in every microplate well was measured. As shown in Fig. 4a, the fluorescence intensity was gradually increased with the increasing concentration of the standard antigen. The standard curve was constructed by plotting the fluorescence intensity (Y) against the CRP sample concentration (X) of analytes. Figure 4b showed that a good linear correlation was obtained with the concentration range from 1.56 to 400 ng/mL and the best fit for the calibration curve was Y = 0.778X + 4.372 with R^2^ = 0.992. The limits of detection (LOD) and quantification (LOQ) were the key parameters for evaluating detection method. Here we tested the black wells (the negative control samples) ten times at the same time, according to the provisions of International Union of Pure and Applied Chemistry (IUPAC), the LOD was calculated as three times the standard deviation (SD) to the slope of the min-concentration calibration plot (LOD = 3 SD/slope) and LOQ was calculated as ten times the same ratio (LOQ = 10 SD/slope). The final result showed that the novel CRP assay had a LOD was 0.46 ng/mL and a LOQ was 1.53 ng/mL.

The developed immunoassay is superior to most of the current analysis techniques for detection of CRP in terms of highly sensitivity [40, 41]. The common clinical laboratory assays used to quantify CRP have a lower detection range in μg/mL, including latex agglutination or commercially available POCT methods. Recent new methodology-based immunoassays, such as sandwich ELISA, fluorescence sandwich immunoassay began to improve sensitivities in the range from μg/mL to ng/mL. Here this new developed FLISA method achieves ng/mL, at the same time, it offers a broader range. In addition, the QDs-mAb probes and the coated microplates could still keep good stability as long as 90 days and its fluorescence intensity maintain around 60% of the original value (shown in Additional file 1: Figure S5).

### Precision and accuracy

It is well known that precision is an important indicator of measuring in vitro diagnosis, including intra-assay and extra-assay of imprecision. Three concentrations of quality control was 10, 50, 200 ng/mL, respectively using three batches of CRP reagent. By measuring each concentration five times, we calculated quality-control serum values of the mean, standard deviation, and coefficient of variation (CV). As shown in Table 2, the results met relevant provision of the test of biological products, intra-assay within CV <10%, and inter-assay within CV <15%. This method possessed excellent stability and repeatability on account of the good intra-assay and the extra-assay.

**Table 2:** Imprecision the intra-assay and the extra-assay

Standard of CRP (ng/mL)	Intra-assay			Extra-assay
	1st batch (CV %)	2nd batch (CV %)	3rd batch (CV %)	Avergae (CV %)
Low	8.29 ± 0.63 (7.67)	7.28 ± 0.72 (9.91)	7.56 ± 0.75 (9.96)	7.50 ± 1.09 (14.5)
Middle	46.57 ± 3.01 (6.45)	48.43 ± 3.64 (7.52)	43.71 ± 2.46 (5.62)	46.24 ± 3.48 (7.54)
High	200.73 ± 18.38 (4.17)	203.35 ± 15.34 (7.54)	193.53 ± 10.79 (5.57)	199.21 ± 11.79 (5.92)

Recovery measurements were performed to assess the overall accuracy of the QD-based assay. The negative serum samples were spiked with a series of known standard CRP antigens for analysis, and the final concentrations covered the low, medium, and high-risk levels. The results shown in Table 3 indicated that all of the recovery rates were within the range of 95.4–105.7%, and the standard deviation was less than 15%. Due to the high accuracy and excellent repeatability, this FLISA method allowed for high-throughput detection on clinical research.

**Table 3:** Recoveries of different concentrations of antigen in human negative serum

Sample	Experimental value (ng/mL)	Theroratical value (ng/mL)	Recovery (%)	RSD (%)
P1	312.7	300	104.2	3.43
P2	146.6	150	97.7	5.81
P3	71.52	75	95.4	5.19
P4	10.57	10	105.7	8.48
P5	5.28	5	105.6	9.07

### Specificity of FLISA

**Fig. 5:**
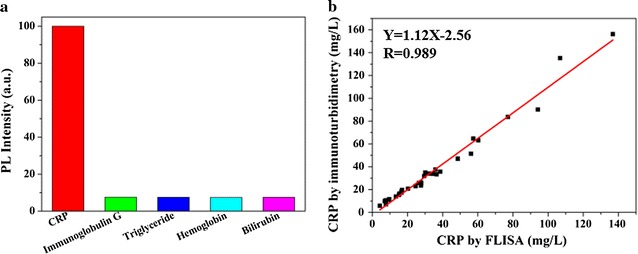
Cross-reactionof FLISA (**a**), and comparison of results for CRP concentrations by FLISA and Roche immunoturbidimetry (**b**)

The interference factors (immunoglobulin G, triglyceride, hemoglobin, and bilirubin) were selected as cross-reaction interference to evaluate the specificity of the FLISA method. As shown in Fig. 5a, the result showed that interference (10 mg/mL of immunoglobulin G, 20 mg/mL of triglyceride, 10 mg/mL of hemoglobin, and 0.35 mg/mL of bilirubin) did not interfere with the immunoassay, and validated that the interference factors did not have specific Ag–Ab combination, which suggested that the assay for a target analyte was not affected by the presence of other interference. The background fluorescence interference was caused by the autofluorescence of specific biomolecules in the serum, which leaded to appear the weak, nonspecific signals. However, it did not affect the accuracy of the CRP detection by introducing a reagent blank control consisting of the negative serum.

### Clinical sample detection and analysis

In this study, we also collected thirty-four CRP human serum samples from Shenzhen Six People’s Hospital for real clinical sample test. The immunoturbidimetry is based on the precipitation reactions of Ag–Ab, the turbidity appears by polyethylene glycol (PEG) and is measured by optical instruments. Due to the good sensitivity and accuracy, the immunoturbidimetry is mostly used for clinical diagnosis method of CRP by many hospitals worldwide. And Roche immunoturbidimetry generally has been recognized as gold standard test in vitro diagnostic benchmark and it is used to refer to the most accurate test possible without restrictions. By comparing the results from Roche immunoturbidimetry and this FLISA, the linear regression equation is Y = 1.12X−2.56 and the highly significant correlation is 0.989 (>0.9) as shown in Fig. 5b, which indicates the FLISA method has excellent concordance as the gold Roche immunoturbidimetry. Therefore, this new established FLISA is able to rapidly analyze serum samples from clinical patients after appropriate dilution with a high sensitivity and accuracy.

## Conclusion

Currently, the majority common assays do not provide high-throughput quantification of multiple analysis and simple operation steps. Therefore, the development of methods for the accurate, sensitive, rapid, and convenient detection is important need in vitro diagnosis. In this report, a simple, rapid, and high sensitivity method by a novel quantum dots-labeled immunosorbent assay has been developed, which only takes half of the analysis time and offers a lower LOD, a wide liner detection range than those of ELISA. The water-soluble QDs as novel fluorescent probe possess excellent stability, without influencing on the performance of antibody protein, and can be applied to combine other antibody or antigen protein. This novel analytical tool can play an important role in preliminary test of CRP protein levels, and provide wide application in clinical research because the assay meets the needs felicitously of the simplicity, sensitivity, and high-throughput with a short time and low cost. Moreover, the FLISA method gives a very promising outlook for lower LOD as well as high-throughput capability which shows the potential for in vitro diagnostic tool in the future.

## Additional file

**Additional file 1.** Additional figures.

## Abbreviations

Ab: antibody
Ag: antigen
BS: sodium borate buffer
BSA: bovine serum albumin
CB: carbonate-bicarbonate buffer
CRP: C-reactive protein
CV: coefficient of variation
CVD: cardiovascular disease
DLS: dynamic light scattering
ELISA: enzyme-linked immunosorbent assay
EDC: 1-ethyl-3-(3-(dimethylamino) propyl) carbodiimide
FLISA: fluorescence-linked immunosorbent assay
HCG: human chorionic gonadotrophin
IUPAC: International Union of Pure and Applied Chemistry
LFIA: lateral flow immunoassay
LOD: the limit of detection
LOQ: the limit of quantification
mAb: monoclonal antibody
MES: 2-(*N*-morpholino) ethanesulfonic acid
ODE: 1-octadecene
PBS: phosphate buffered saline
PBS-T: 0.01 M PBS contains 0.05%(v/v) Tween-20
PEG: polyethylene glycol
PL: photoluminescence
PMAH: polymaleic acid n-hexadecanol ester
POCT: point-of-care testing
QDs: quantum dots
QDs-mAb: quantum dots labeled monoclonal antibody
R: the correlation coefficient value
SD: standard deviation
sulfo-NHS: *N*-hydroxysulfosuccinimide
