# Supplementary material for: Highly sensitive and accurate detection of C-reactive protein by CdSe/ZnS quantum dot-based fluorescence-linked immunosorbent assay
Source: J Nanobiotechnology. 2017 May 2;15:35. doi: 10.1186/s12951-017-0267-4 (PMC5414212; doi:10.1186/s12951-017-0267-4)
Supplement: Supplementary file 1 — Additional file 1. Additional figures. [file 12951_2017_267_MOESM1_ESM.docx]

**Supporting Information**

ree﷽﷽﷽﷽﷽﷽﷽﷽mmunosorbent assay **Highly sensitive and accurate detection of C-reactive protein by CdSe/ZnS quantum dot-based fluorescence-linked immunosorbent assay**

Yanbing Lv^1^, Ruili Wu^1,*^, Kunrui Feng^2^, Jinjie Li^1^, Qing Mao^2^, Hang Yuan^2^, HuaibinShen^1^, Xiangdong Chai^2,*^, and Lin Song Li^1,*^

*^1^Key Laboratory for Special Functional Materials of Ministry of Education, Henan University, Kaifeng 475004, China*

*^2^NepQD Biotech Corp, Taizhou 225300, China*

1. **Synthesis of hydrophobic CdSe/ZnS PL QDs**

Precursor Preparation: Se (0.188 g, 2.4 mmol), OA (2 g, 7.2 mmol), and 17.5 mL of ODE were loaded in a 50 mL three-neck flask and degassed, the mixture was heated to 100 ºC then maintained for 20 min and subsequently heated to 220 ºC then maintained for 3 h. Zinc precursor was prepared by dissolving Zn(Ac)_2_ (0.8 g, 4.4 mmol) in OA (10.83 g, 38.98 mmol) and 43.20 mL of ODE, and heating to 120 ºC under a vacuum for 2 h.

Synthesis of CdSe QDs: A mixture (4 g in total) of CdO (0.0154 g, 0.12 mmol), oleic acid (0.36 mmol), and ODE was loaded in a 25 mL three-neck flask and heated to 240 ºC under nitrogen flow to obtain a clear colorless solution. When it was heated to 280 ºC, 2 mL (0.24 mmol) Se stock solution was injected into the flask. Aliquots were taken at different time intervals, UV-vis and PL spectra were recorded for each aliquot. When the targeted size of nanocrystals was obtained, the reaction mixture was allowed to cool down to room temperature.

Synthesis of CdSe/ZnS QDs: 3 mL of ODE and 1.0 g of OA were loaded into a 25 mL reaction vessel. The CdSe QDs in hexanes (2.7×10^−7^ mol) were added, and the system was maintained at 100 ºC under N_2_ flow for 30 min to remove hexanes and other undesired materials. The solution was heated to 160 ºC under N_2_ flow for the growth of ZnS shell. At 180, 200, 220, 240, and 250 ºC, the Zn and S precursors (Zn precursor was prepared by dissolving ZnO in the mixture of OA and ODE at 310 ºC while S precursor by dissolving sulfur in ODE at 150 ºC) with calculated amounts were added, respectively. After the reaction was completed, the temperature was cooled down to room temperature.





**Fig. S1.** Absorption and PL spectra of the as-synthesized CdSe/ZnS core/shell QDs in chloroform.

1. **Synthesis of amphiphilic oligomer**

A typical procedure to synthesize amphiphilic oligomer (polymaleic acid *n*-hexadecanolester, PMAH) was as follows: 0.98 g of PMA (0.01 mol MA monomer) and 0.81 g of *n*-hexadecanol (3.3×10^-3^ mol) were dissolved in 100 mL anhydrous acetone. To synthesize amphiphilic oligomer, the mixture required 2 mL alkaline catalysis (triethylamine) and refluxed at 57 °C for 48 h. The obtained solution was treated with rotary evaporation to remove most of acetone, and PMAH was precipitated by adding excess of anhydrous toluene.

1. **Synthesis of water-soluble CdSe/ZnS QDs**

The PMAH have hydrophobic chains available for anchoring the hydrophobic CdSe/ZnS QDs and free carboxylic acid groups available for further surface modification. The hydrophobic QDs and PMAH bound to each other via the phase-transfer stem to form PMAH-stabilized PL QDs. First, 3.61 g of PMAH (0.2 mmol) was dissolved in 20 mL of chloroform. Subsequently, 2 mL of CdSe/ZnS QDs (0.04 mmol, dispersed in chloroform) was added to the PMAH solution, which was then stirred for 24 h (room temperature) in a closed container. The chloroform was then slowly evaporated by rotary evaporation, and the remaining QD film was dispersed in ammonia water (pH 9.0) with sonication. The solutions were passed through a 0.22 μm Nylon syringe filter, washed, and then centrifuged four times at 20,000 g to remove excess oligomers.

**

**

**Fig. S2.** PL spectra of the as-synthesized CdSe/ZnS core/shell QDs in chloroform and the corresponding water-soluble QDs in water.

1. **Zeta potential of aqueous CdSe/ZnS QDs and QDs-mAb**





**Fig. S3.** Zeta potential of the aqueous CdSe/ZnS QDs and QDs-mAb.

1. **Screening the QD-mAb diluted buffer**


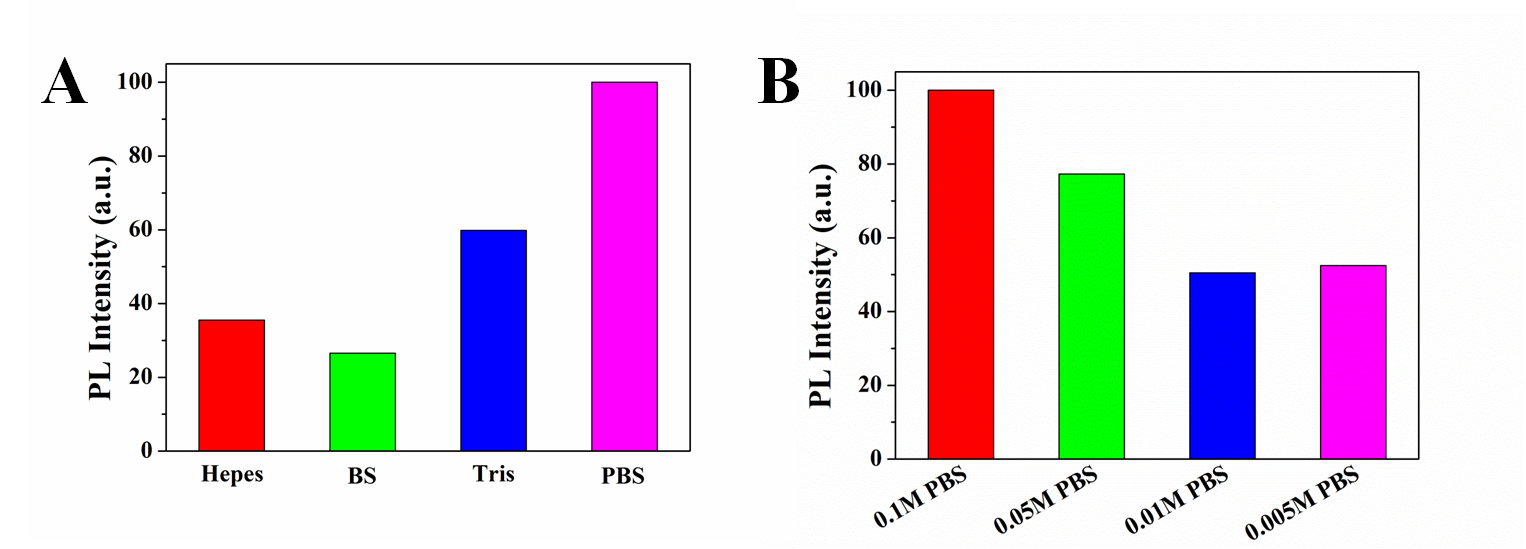


**Fig. S4.** The PL intensity of the FLISA results when QD-mAb in different buffer (A) and in PBS buffer at different ionic strengths (B), the CRP antigen concentration is 100 ng/mL.

The QD-mAb was diluted in different buffers, including 0.01M Hepes (pH 8.0), 0.01M BS (pH 8.0), 0.01M Tris (pH 8.0), and 0.01M PBS (pH 7.4) (both contain 10% calf serum). As shown in Fig. S3A, it had a better specific Ag-Ab combination when the QD-mAb in PBS buffer. Subsequently, the fluorescence intensity was influenced in the PBS buffer at different ionic strengths. In Fig. S3B, it indicates that the max fluorescence intensity was in 0.1M PBS. At last, 10% calf serum (v/v) in 0.1M PBS (pH 7.4) was chosen as the probe diluted buffer.

1. **The stability of the** **QDs-mAb probes and the coated microplates**

**

**

**Fig. S5.** The stability experiment of high concentration (200 ng/mL), medium concentration (50 ng/mL), low concentration (5 ng/mL) (The microplate with coating antibody stored at 4 degree).

In order to examine the stability of the QDs-mAb probes, we coated a group of microplates with CRP antibody, which to prove the QDs-mAb probes are still reliable enough to quantitatively detect CRP in a storage period of 90 days. We choose to quantitatively detect CRP antibody with high (200 ng/mL), medium (50 ng/mL), low (5 ng/mL) concentrations. The results shown in Fig.S5 indicate that the high concentration fluorescence intensity still can maintain 60% of the original PL intensity after 90 days (stored at 4 degree). Besides, the medium (50 ng/mL) and low (5 ng/mL) concentrations are met good stability.
